# Differences in Transporters Rather than Drug Targets Are the Principal Determinants of the Different Innate Sensitivities of *Trypanosoma congolense* and *Trypanozoon* Subgenus Trypanosomes to Diamidines and Melaminophenyl Arsenicals

**DOI:** 10.3390/ijms23052844

**Published:** 2022-03-05

**Authors:** Marzuq A. Ungogo, Gustavo D. Campagnaro, Ali H. Alghamdi, Manal J. Natto, Harry P. de Koning

**Affiliations:** 1 Institute of Infection, Immunity and Inflammation, College of Medical, Veterinary and Life Sciences, University of Glasgow, Glasgow G12 8QQ, UK; 2226184u@student.gla.ac.uk (M.A.U.); campagnarogd@gmail.com (G.D.C.); aayfan@bu.edu.sa (A.H.A.); manal.natto@glasgow.ac.uk (M.J.N.); 2 Department of Veterinary Pharmacology and Toxicology, Ahmadu Bello University, Zaria 810107, Kaduna State, Nigeria

**Keywords:** *Trypanosoma congolense*, *Trypanosoma evansi*, cymelarsan, pentamidine, suramin, diminazene, drug transporter, TbAT1, TbAQP2, MFST

## Abstract

The animal trypanosomiases are infections in a wide range of (domesticated) animals with any species of African trypanosome, such as *Trypanosoma brucei*, *T. evansi*, *T. congolense*, *T. equiperdum* and *T. vivax*. Symptoms differ between host and infective species and stage of infection and are treated with a small set of decades-old trypanocides. A complication is that not all trypanosome species are equally sensitive to all drugs and the reasons are at best partially understood. Here, we investigate whether drug transporters, mostly identified in *T. b. brucei*, determine the different drug sensitivities. We report that homologues of the aminopurine transporter TbAT1 and the aquaporin TbAQP2 are absent in *T. congolense*, while their introduction greatly sensitises this species to diamidine (pentamidine, diminazene) and melaminophenyl (melarsomine) drugs. Accumulation of these drugs in the transgenic lines was much more rapid. *T. congolense* is also inherently less sensitive to suramin than *T. brucei*, despite accumulating it faster. Expression of a proposed suramin transporter, located in *T. brucei* lysosomes, in *T. congolense*, did not alter its suramin sensitivity. We conclude that for several of the most important classes of trypanocides the presence of specific transporters, rather than drug targets, is the determining factor of drug efficacy.

## 1. Introduction

Animal African trypanosomiasis (AAT) is a diverse complex of diseases caused by African trypanosome species, including (but not limited to) *Trypanosoma brucei brucei*, *T. congolense*, *T. vivax*, *T. evansi* and *T. equiperdum* [1]. In sub-Saharan Africa, animal trypanosomiasis is mostly transmitted by tsetse flies and the etiological agents are mostly *T. b. brucei*, *T. b. rhodesiense* (Subgenus *Trypanozoon*), *T. vivax* (Subgenus *Duttonella*) and *T. congolense* (Subgenus *Nannomonas*). Trypanosomiasis has a severe impact on livestock rearing on the continent, with infections in ruminants, equines, pigs and dogs being common. Outside the tsetse belt, AAT is caused by *T. vivax* and *T. evansi*, which can be transmitted by other biting insects, as well as by *T. equiperdum*, which is sexually transmitted between horses, giving rise to a wasting disease called dourine [2]. *T. vivax* is a significant pathogen for a broad range of animals in South America and Africa [3,4,5] but has only very recently been reported for the first time in Asia [6]. In contrast, *T. evansi* has long been known to be an important veterinary infection and of all the pathogenic *Trypanosoma* spp. has the widest geographical distribution, from Africa to South America, Asia, Indonesia and the Philippines [7,8,9,10].

The total amount of damage done by AAT to (often developing) economies and to agricultural production is hard to tally up, but the yearly cost of bovine trypanosomiasis in Africa alone has been estimated as approximately US$ 5 billion [11]. Control of this disease spectrum is very difficult, considering the diversity of transmission modes and vectors, the enormous geographical spread, the lack of any vaccine and the challenges of diagnosis. In practice, chemotherapeutic treatment with veterinary trypanocides is usually the only option available but this has many challenges, also. All the available drugs are decades old, resistance to all drugs has been reported, the different species have different levels of sensitivity to the various drugs, several drugs are poorly tolerated by some of the important host species, some drugs, such as quinapyramine, induce cross-resistance to other drugs, and availability and quality can be poor [1]. Here we will focus on the innate differences in drug sensitivity of different AAT-causing trypanosome species, particularly *Trypanozoon* subgenus species *T. b. brucei* and *T. evansi* and *Nannomonas* species *T. congolense*. We recently highlighted that *T. congolense* is far less susceptible than *T. b. brucei* to the diamidine trypanocides diminazene and pentamidine and suggested that differences in drug accumulation may be responsible for this important difference. Suramin, used since 1920 for East African trypanosomiasis [12], is often the drug of choice to treat *T. evansi* infections in camels, known as surra, although melarsen oxide cysteamine (melarsomine, Cymelarsan, MelCy), introduced in the mid-1980s, is an efficacious alternative [13]. However, melarsomine and suramin are not considered effective against animal trypanosomiasis in sub-Saharan Africa [1]. Indeed, *T. congolense* and *T. vivax* are both believed not to be very susceptible to suramin in vivo [14], but little evidence can be found in the literature. 

The action of anti-trypanosomiasis drugs has been predominantly investigated in *T. b. brucei*, which has always been considered a convenient model organism. For most of the traditional trypanocides, the activity and, frequently, the selectivity over human cells depends on the efficient uptake of the drugs by the parasite [15]. Pentamidine is taken up by the TbAT1/P2 aminopurine transporter [16,17] as well as by two additional transporters, initially named high affinity pentamidine transporter (HAPT1) and low affinity pentamidine transporter (LAPT1) [18]. HAPT1 was subsequently identified as an aquaglyceroporin, TbAQP2 [19,20,21], but the identity of the low-affinity carrier has not been elucidated. Diminazene, while a diamidine like pentamidine, is taken up solely by the TbAT1/P2 aminopurine transporter [22], as is the furamidine class of diamidines [23]. The lack of passage through TbAQP2 is explained by the short, inflexible linkers of diminazene and furamidine [20]. In contrast, the melaminophenyl arsenicals, melarsoprol and the derived veterinary product melarsomine, have been shown to be internalised by both TbAT1/P2 [24,25] and HAPT1/TbAQP2 [18,20,21]. The activity of eflornithine (α-difluoromethyl ornithine) is likewise dependent on a *T. brucei* carrier, amino acid transporter TbAAT6 [26,27,28]. Similarly, suramin is taken up specifically into *T. b. brucei* by receptor-mediated endocytosis, after binding to cell surface receptors, predominantly ISG75 [29,30], and isometamidium is efficiently taken up into trypanosomes by an as yet unidentified transporter [31,32]. Only the nitro compounds nifurtimox and fexinidazole are believed to enter trypanosomes by simple diffusion, although this likely only signifies that no serious work has as yet been done on the uptake of such compounds [33]. Interactions of nifurtimox with transporters at the blood–brain barrier have been reported [34,35] and a point mutation in a *T. brucei* transporter, Tb9.211.2900, has been reported in both nifurtimox-resistant and fexinidazole-resistant cells [36]. 

In view of the important role transporters have in determining the sensitivity of *T. brucei* to the current trypanocidal drugs, we here explore whether expression of genes involved in drug uptake in brucei-group trypanosomes would sensitise *T. congolense* to these compounds.

## 2. Results and Discussion

### 2.1. Sensitivity of Different Trypanosoma Species to Standard Trypanocides

**Figure 1:**
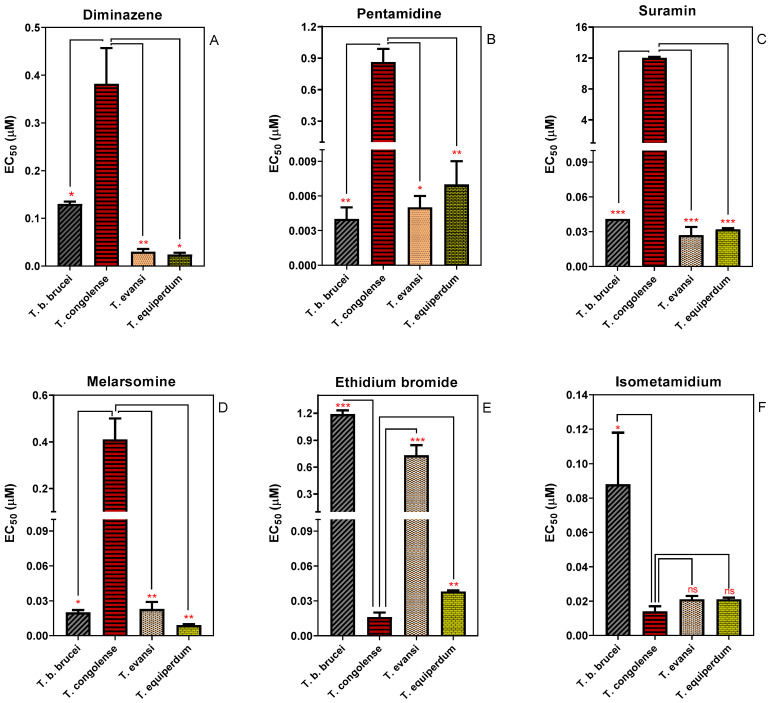
Sensitivity of four species of animal trypanosomes to trypanocides. (**A**) Diminazene; (**B**) Pentamidine; (**C**) Suramin; (**D**) Cymelarsan; (**E**) Ethidium bromide; and (**F**) Isometamidium. Sensitivity is represented as EC_50_ averages of 3–5 independent determinations (mean ± SEM). * *p* < 0.05; ** *p* < 0.01; *** *p* < 0.001 by unpaired Student’s *t*-test.

The Alamar Blue (resazurin) assay has been used to characterise the drug sensitivity profile of different Trypanosoma species [37,38,39,40]. We used the assay to determine and compare the in vitro sensitivity of four species of animal trypanosomes (Figure 1). *T. congolense* displayed a significantly (*p* ˂ 0.05) lower sensitivity to diminazene aceturate, pentamidine, melarsomine and suramin than any of the brucei group (Trypanozoon) trypanosomes, *T. b. brucei*, *T. evansi* and T. equiperdum. Specifically, the EC_50_ values of diminazene aceturate (a diamidine) and melarsomine (a melaminophenyl arsenical) in *T. congolense* were 3- and 20-fold higher than in *T. brucei*, respectively. Moreover, *T. congolense* was 200 times less sensitive to pentamidine and 296 times more resistant to suramin relative to *T. brucei* (Figure 1). However, *T. congolense* was significantly more sensitive to the phenanthridine compound ethidium bromide compared to the other species investigated here. Although *T. congolense* also trended towards higher sensitivity to the other phenanthridine compound, isometamidium, this did not reach statistical significance when compared with *T. equiperdum* and *T. evansi*. It has been widely reported that in brucei group trypanosomes drug sensitivity and resistance is often linked to the presence or absence of particular transport proteins, such as TbAT1, TbAQP2, TbMFST and TbAAT6, which mediate the uptake of diamidines and melaminophenyl arsenicals (TbAT1 and TbAQP2), suramin (TbMFST) and eflornithine (TbAAT6), respectively [26,29,41]. However, the now well-established models of drug transport and resistance do not seem to apply to all African trypanosome species, as recently shown for diminazene uptake and resistance in *T. congolense* [42]. We here investigate whether the relatively low sensitivity of *T. congolense* for diamidines, melaminophenyl arsenicals and suramin is linked to the absence of orthologues of TbAT1, TbAQP2 and TbMFST. 

As shown in Table 1, such orthologous proteins can readily be identified in *T. evansi* and *T. equiperdum* (species that are highly sensitive to these drugs (Figure 1)) but not in *T. congolense*. The TbAT1 orthologue in *T. evansi* was first amplified by Witola et al. [43] and is 99.35% identical to that of *T. brucei* (GenBank accession number AGT37292.1). BLAST searches using TbAT1 sequences in *T. equiperdum* genome found a gene (Genbank SCU70586.1) with approximately 99.8% identity. *T. equiperdum* AT1 has previously shown similar functional characteristics to TbAT1 [38,44]. BLAST searches with TbAQP2 found an orthologue with 100% identity in *T. evansi*, TevSTIB805.10.14910, annotated as TevAQP9. An identical AQP2 sequence was also found in *T. equiperdum* strain STIB818 [45]. 

The *T. brucei* lysosomal MFST transporters exist as an array of three non-identical tandem copies on chromosome 9, each of which have protein orthologues in both *T. evansi* and *T. equiperdum* with at least 96% amino acid sequence identity (Table 1). However, no orthologue of TbAT1, TbAQP2 or TbMFST, or indeed any gene with >75% nucleotide sequence identity to either of them, was found in the *T. congolense* genome (nor in the *T. vivax* genome). We tested the hypothesis that the lack of these transporters renders *T. congolense*, at least, less susceptible to diamidines, melaminophenyl arsenicals and suramin by expressing each of these transporters separately in lab-adapted strain TcoIL3000.

### 2.2. Cloning of T. brucei Drug Transporters and Their Expression in T. congolense

**Figure 2:**
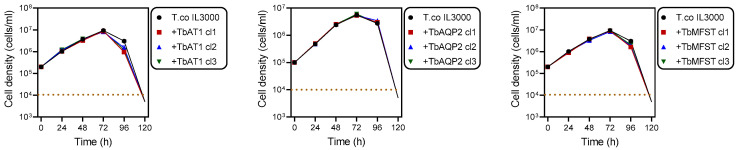
Growth curves of *T. congolense* IL3000 and the same strain expressing *TbAT1*, *TbAQP2* or *TbMFST*. The dotted line indicates the detection limit for trypanosome counting by haemocytometer counting.

TbAT1, TbAQP2 or TbMFST were each amplified from *T. brucei* genomic DNA, purified and separately ligated into a digested pMPB-DP-012 plasmid or its SalI site-mutated derivative. Primers for the amplification of TbAT1 and TbMFST were designed to eliminate the stop codon and fuse the ORF to a C-terminal 6x haemagglutinin (HA) tag. The constructed plasmid was linearised with Asc1 and transfected into *T. congolense* and its correct integration verified by PCR. Positive clones were selected by adding 0.5 μg/mL blasticidin to the growth medium and characterised using drug sensitivity and uptake assays. From the blasticidin selection medium, multiple clonal lines were generated, and for each construct three clonal lines were used for subsequent experiments. The expression of these transporters had no effect on growth under standard in vitro conditions (Figure 2).

### 2.3. Uptake of Drugs in Different Trypanosome Species—Influence of TbAT1 and TbAQP2

#### 2.3.1. Pentamidine

**Figure 3:**
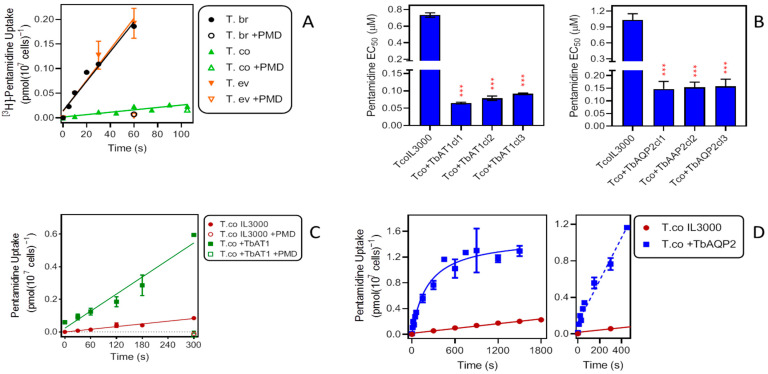
Effects of TbAT1 and TbAQP2 on pentamidine (PMD) uptake and sensitivity in *T. congolense*. (**A**) Uptake of 0.025 µM [^3^H]-pentamidine by bloodstream forms of *T. b. brucei* (*n* = 4), *T. evansi* (*n* = 3) and *T. congolense* (*n* = 4). Data shown are the average of three or four experiments combined; each experiment was performed in triplicate; lines are linear regressions calculated by Prism 9 (r^2^ = 0.97, 0.97 and 0.85, respectively), all with slope significantly non-zero (F test, *p* < 0.05) and not significantly non-linear (runs test, *p* > 0.3). +PMD, addition of 1 mM unlabelled pentamidine to the assay buffer. (**B**) Effects of expression of TbAT1 (left panel) or TbAQP2 (right panel) in *T. congolense* IL3000 (TcoIL3300) on sensitivity to pentamidine (PMD). Bars are the average ± SEM of three independent determinations, for three different clonal populations (cl1–cl3), arising from different transfections with the gene of interest. NS, not significantly different from untransfected control; *** *p* < 0.0001 by unpaired *t*-test. (**C**) Uptake of 0.025 µM [^3^H]-pentamidine by bloodstream forms of *T. congolense* IL3000 (untransfected and expressing *TbAT1*). The data shown are the average ± SEM of two experiments, each performed in triplicate. Lines were calculated by linear regression (r^2^ = 0.97 and 0.96, resp.) and not significantly non-linear (*p* > 0.3); slopes were significantly non-zero (*p* < 0.001). +PMD, addition of 1 mM unlabelled pentamidine to the assay buffer. (**D**) Uptake of 0.025 µM [^3^H]-pentamidine by bloodstream forms of *T. congolense* IL3000 (untransfected and expressing TbAQP2). The data shown are the average ± SEM of four experiments each performed in triplicate. Lines were calculated by linear regression (IL3000, r^2^ = 0.98) and non-linear regression (+TbAQP2). For the +TbAQP2 uptake, a linear regression was also performed over the interval 0–450 s (dashed line, r^2^ = 0.97, slope significantly non-zero (*p* < 0.0001), not significantly non-linear (*p* = 0.5).

We have previously reported on the rate of pentamidine uptake in *T. b. brucei*. In *T. b. brucei* bloodstream forms isolated from infected Wistar rats, a rate of 0.0045 ± 0.0004 pmol(10^7^ cells)^−1^s^−1^ was obtained for 25 nM [^3^H]-pentamidine [46]. Here, we found an average value of 0.0030 ± 0.0003 pmol(10^7^ cells)^−1^s^−1^ (*n* = 4; Figure 3A) for the same strain and radiolabel concentration—slightly lower because the TbAT1/P2 transporter is more highly expressed in ex vivo cells [23]. These rates were compared with those observed in cultured bloodstream forms of *T. evansi* (0.0031 ± 0.0004 pmol(10^7^ cells)^−1^s^−1^; *n* = 3) and *T. congolense* (0.00024 ± 0.00004 pmol(10^7^ cells)^−1^s^−1^; *n* = 4), performed in parallel (Figure 3A). Whereas pentamidine uptake by *T. evansi* was not significantly different from that in *T. b. brucei* (*p* = 0.76; F-test comparing linear regression lines; Prism 9), the rate displayed by *T. congolense* was more than an order of magnitude lower (*p* < 0.0001). 

Upon expression of TbAQP2 or TbAT1 in *T. congolense* IL3000, sensitivity to pentamidine was highly significantly increased in each of three independent clones. For +TbAT1 cells, the EC_50_ changed from 0.73 ± 0.03 µM in IL3000 control cells to an average of 0.078 ± 0.008 µM (*p* < 0.001). In a separate series of experiments the expression of TbAQP2 similarly reduced the EC_50_ from 1.03 ± 0.12 µM to 0.15 ± 0.003 µM (*p* < 0.001) (Figure 3B). Transport of 0.025 µM [^3^H]-pentamidine into the transgenic *T. congolense* was much increased. Transport in the +TbAT1 cells increased 6.3-fold, from 0.00028 ± 0.00003 pmol(10^7^ cells)^−1^s^−1^ to 0.0017 ± 0.0002 pmol(10^7^ cells)^−1^s^−1^ (*p* <0.0001) (Figure 3C). Pentamidine uptake was similarly increased upon expression of TbAQP2 in IL3000. Figure 3D shows an initial linear phase of uptake followed by a plateau after 450 s. Using linear regression over that initial phase to compare uptake of [^3^H]-pentamidine in control cells, it was found to be increased over 18-fold (0.0023 ± 0.0002 versus 0.00012 ± 0.00001 pmol(10^7^ cells)^−1^s^−1^; *p* < 0.0001). It therefore appears that the rate of influx of pentamidine into *T. congolense* limits this parasite’s sensitivity to the drug and that expression of the known *T. brucei* pentamidine transporters both increases the uptake rate and the sensitivity to pentamidine. With either drug transporter expressed in *T. congolense* IL3000, both parameters remained somewhat below the observed level for *T. brucei* bloodstream forms, and it may be that full sensitivity would require the expression of both at the same time.

#### 2.3.2. Diminazene Aceturate (DA)

**Figure 4:**
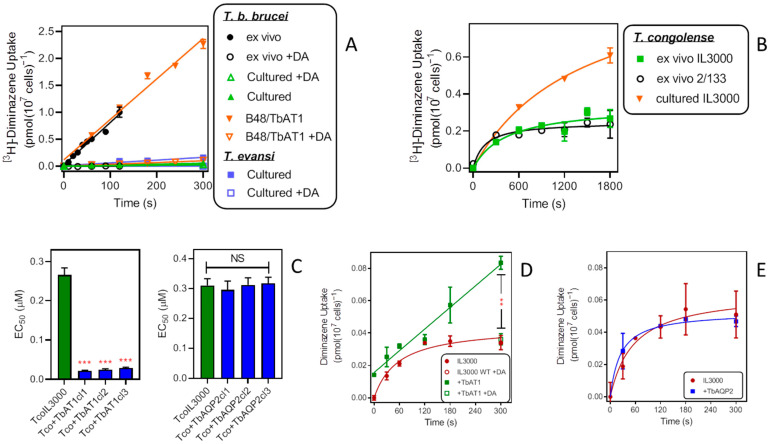
Effects of TbAT1 and TbAQP2 on diminazene aceturate (DA) uptake and sensitivity by *T. congolense*. (**A**) Uptake of 0.1 µM [^3^H]-diminazene (DA) in cultured and ex vivo *T. brucei* and cultured *T. evansi* bloodstream forms. All lines were calculated by linear regression with r^2^ > 0.95. +DA indicates the addition of 1 mM DA to the assay buffer. In the absence of excess DA all slopes were significantly different from zero (*p* < 0.01) and not significantly non-linear (*p* > 0.3). All symbols are average ± SEM of triplicate determinations. (**B**) Uptake of 0.1 µM [^3^H]-DA in cultured and ex vivo *T. congolense*. Lines were calculated by non-linear regression. All symbols are averages ± SEM of triplicate determinations. (**C**) Sensitivity of *T. congolense* IL3000 (green bars) and TcoIL3000 expressing either TbAT1 or TbAQP2 to DA. Bars show averages ± SEM of three independent EC_50_ determinations. *** *p* < 0.001 by *t*-test; NS, not significant. (**D**) Uptake of 0.2 µM [^3^H]-DA in cultured IL3000 or the same cells expressing TbAT1, lines being calculated by non-linear and linear regression (r^2^ = 0.98), respectively. All symbols are averages ± SEM of triplicate determinations. ** *p* < 0.01. (**E**) Like D but with IL3000 expressing *TbAQP2*.

It has been previously established that uptake of diminazene is essentially mediated by TbAT1/P2 only in *T. brucei* bloodstream forms [22,39], *T. evansi* [43] and *T. equiperdum* [38]. However, the issues with the variable expression levels of TbAT1 [23] complicate a quantitative comparison between AAT trypanosome species. This is illustrated in Figure 4A: [^3^H]-diminazene uptake in wild-type *T. b. brucei* freshly isolated from a rat was rapid (0.0079 ± 0.0004 pmol(10^7^ cells)^−1^s^−1^) and virtually identical to uptake in a *T. b. brucei* cell line stably expressing *TbAT1* from vector pHD1336 [47], B48/TbAT1 (0.0075 ± 0.0006 pmol(10^7^ cells)^−1^s^−1^; *p* = 0.71). In contrast, the same wild-type *T. b. brucei* grown in culture took up diminazene very poorly (0.00018 ± 0.00001 pmol(10^7^ cells)^−1^s^−1^; *p* < 0.0001). In cultured *T. evansi* bloodstream, to the extent that a quantitative comparison is meaningful, the rate was similar though somewhat above the cultured *T. b. brucei* (0.00052 ± 0.00006 pmol(10^7^ cells)^−1^s^−1^; *p* = 0.0039) (Figure 4A). *T. congolense* is known not to have a functional homolog of the TbAT1 transporter, with the gene known as TcoAT1 encoding a P1-type purine nucleoside transporter instead, the orthologue of TbNT10 [47]. Figure 4B shows that, in contrast to *T. b. brucei*, diminazene uptake from freshly isolated strains is not higher but in fact generally trends lower than with cultured IL3000 bloodstream forms. We have recently reported that [^3^H]-diminazene uptake in *T. congolense* is slow and low affinity, and that linearity of uptake is hard to achieve [42]. It is clear that under comparable conditions (ex vivo, in vitro culture) [^3^H]-diminazene uptake in *T. b. brucei* is much more robust than in *T. congolense*.

Expression of *TbAT1* in *T. congolense* greatly increased sensitivity to diminazene in three independent clonal lines (*p* < 0.001), whereas the expression of *TbAQP2* had no effect on diminazene sensitivity (Figure 4C). Uptake of 0.2 µM [^3^H]-diminazene was linear in +TbAT1 cells, in contrast to the quick plateauing consistently seen in untransfected IL3000 control cells, and was significantly increased by 300 s (*p* < 0.01) (Figure 4D). In the presence of 250 µM unlabelled diminazene aceturate, uptake in the +TbAT1 cells was identical in both cell lines, showing that the TbAT1-mediated flux was saturated by this ligand concentration but the endogenous *T. congolense* uptake system was not. These observations are consistent with previous observations of diminazene uptake in *T. brucei* and *T. congolense* [22,42]. In IL3000 cells expressing TbAQP2 there was no change in the rate of diminazene uptake (Figure 4E).

#### 2.3.3. Suramin

The uptake of suramin, a large molecule that carries six negative charges, across the plasma membrane has long been speculated to involve endocytosis rather than a trans-membrane transporter [29,48]. Recently, it was described that suramin uptake involves binding to the invariant surface protein ISG75, followed by endocytic delivery to the lysosome [28,30]. We have also shown that reduced endocytic activity in *T. brucei* is associated with a similar reduction in the uptake of suramin but not of pentamidine [20].

**Figure 5:**
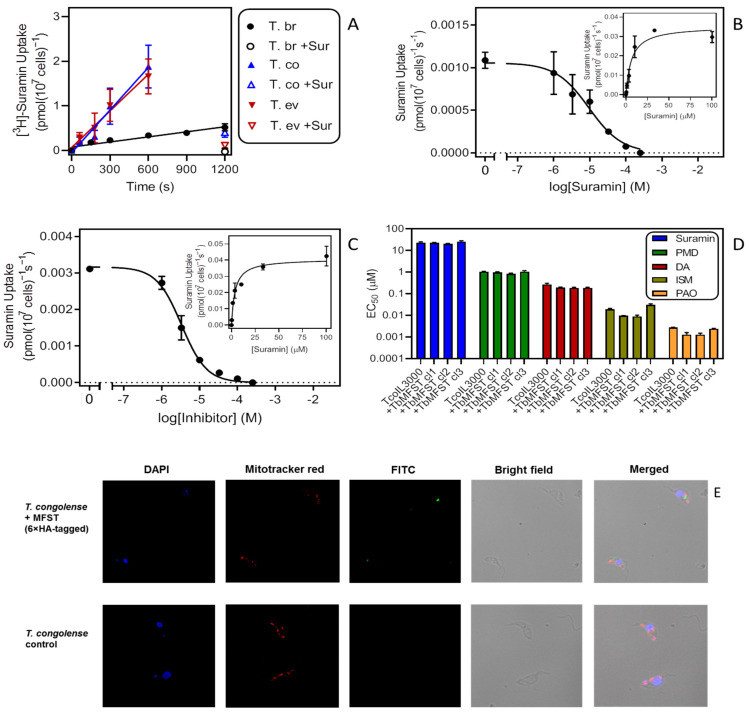
An investigation of suramin uptake and sensitivity in animal trypanosomes. (**A**) Uptake of 0.2 µM [^3^H]-suramin by bloodstream forms of *T. b. brucei*, *T. evansi* and *T. congolense*. The data shown are the averaged results (±SEM) of four (Tbb, Tco) or three (Tev) independent experiments each performed in triplicate. Lines were calculated using linear regression (Prism 9), yielding r^2^ of 0.93 (Tbb), 0.98 (Tev) and 0.97 (Tco). The lines were not significantly non-linear (runs test, *p* > 0.3) and the slopes significantly non-zero (*p* < 0.01). +Sur, addition of 100 µM unlabelled suramin in the assay buffer. (**B**) Uptake of 0.2 µM [^3^H]-suramin by *T. b. brucei* in the presence or absence of variable concentrations of unlabelled suramin. Incubation time was 600 s. Symbols show averages ± SEM of triplicate determinations; a representative experiment of three repeats is shown. Inset: conversion of the inhibition data to a Michaelis–Menten saturation plot. (**C**) Like frame B but with TcoIL3000. (**D**) Sensitivity of TcoIl3000 and three clones of Il3000 expressing TbMFST to suramin, pentamidine (PMD), diminazene aceturate (DA), isometamidium chloride (ISM) and phenylarsine oxide (PAO). Bars represent averages ± SEM of three independent determinations. Drug sensitivity was not significantly different in the untransfected control and the cells expressing TbMFST (*p* > 0.05). (**E**). Immunofluorescence microscopy of *T. congolense* *IL3000* WT (control) and the same cells expressing *TbMFST* (Tb927.9.6360) coupled to a 6×HA tag. Cells were stained for DNA with DAPI, for their mitochondrion with mitotracker red and with FITC-coupled anti-HA antibodies.

Here, we monitored 0.2 µM [^3^H]-suramin uptake by bloodstream forms of *T. b. brucei*, *T. evansi* and *T. congolense* for a side-by-side evaluation. The result with *T. b. brucei* was consistent with our earlier report showing linear uptake of suramin over 20 min [30], with a rate of 0.00038 ± 0.00005 pmol(10^7^ cells)^−1^s^−1^ (*n* = 4) (Figure 5A). Considering the insensitivity of *T. congolense* to suramin, we were surprised to find that this species accumulates suramin almost ten times faster than *T. brucei*, at 0.0032 ± 0.0003 pmol(10^7^ cells)^−1^s^−1^ (*p* < 0.0001; *n* = 4). *T. evansi* displayed a rate (0.0027 ± 0.0002 pmol(10^7^ cells)^−1^s^−1^, *n* = 3) that was not significantly different from the rate of *T. congolense* (*p* = 0.26). For each of the species, 0.2 µM suramin uptake was strongly inhibited by 100 µM unlabelled suramin, showing that the binding was specific and saturable. 

The *K*_m_ values measured for this uptake in the linear phase, presumably reflecting binding to ISG75, were not significantly different for *T. b. brucei* and *T. congolense* (5.87 ± 1.52 µM (*n* = 3) and 6.05 ± 2.39 µM (*n* = 2), respectively; *p* = 0.95, unpaired *t*-test) (Figure 5B,C). This seems to indicate that the interaction of suramin with the cell surface receptor was similar for these species. We hypothesised that a possible explanation for the disparate suramin sensitivity and transport rates could be the proposed escape route of suramin from the lysosome into the cytoplasm [28,29] via a major facilitator superfamily transporter (MFST) located in the lysosomal membrane, which, when knocked down by RNAi, is associated with suramin resistance in *T. brucei*. Our reasoning was that suramin appeared to be taken up and accumulate well in *T. congolense* but without the TbrMFST would remain in the lysosome. This would explain why *T. congolense* is highly resistant to the drug. Moreover, if we consider its multi-target actions in trypanosomes once it is present free in the cytosol [28,30], the difference between the trypanosome species is unlikely to be the result of a difference in a single intracellular drug target. Expression of the *T. b. brucei* MFST in *T. congolense* might therefore allow the efflux of suramin from the *T. congolense* lysosome into its cytosol, becoming sensitive. However, expression of 6xHA-tagged TbMFST had no effect on the sensitivity of IL3000 to any of the drugs tested: suramin, pentamidine, diminazene, isometamidium and phenylarsine oxide (PAO) (Figure 5D). 

Unfortunately, this negative result is open to diverse interpretations, including non-expression or incorrect localization of TbMFST in *T. congolense*. To test for this possibility, we performed fluorescence microscopy with DAPI for DNA, MitoTracker Red for the mitochondrion and FITC-coupled anti-HA antibodies for the localization of TbMFST. Figure 5E shows that the anti-HA signal was limited to a distinct and defined cytosolic location that did not co-localize with the DNA stain and was not inside the mitochondrion. Instead the location of the dot-like signal, although not definitive, was compatible with association with the lysosome, which is positioned between the flagellar pocket and the nucleus. Lacking a known function or phenotype for TbMFST other than in suramin sensitivity, we were unable to come to a conclusion whether the low suramin sensitivity of *T. congolense* could be attributable to differences in the MFST protein or its localisation.

#### 2.3.4. Melarsomine (Cymelarsan; MelCy)

**Figure 6:**
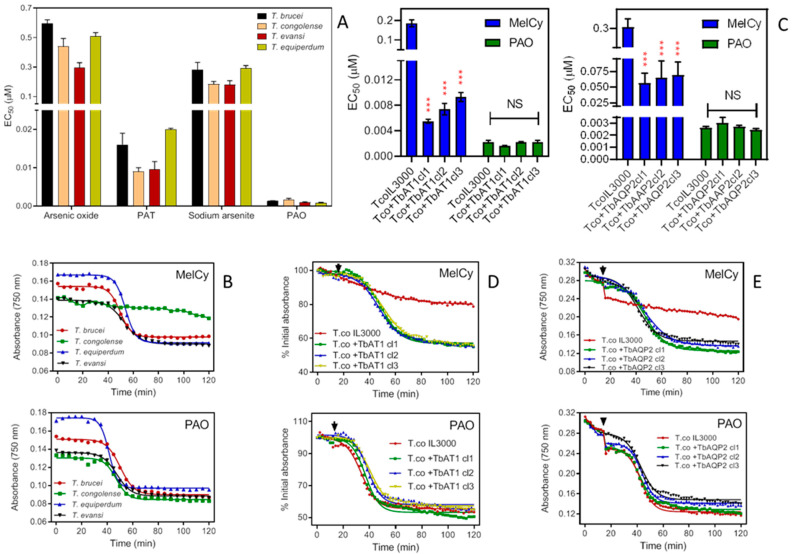
Effects of arsenicals on various *Trypanosoma* species. (**A**) Sensitivity of four species of animal trypanosomes to arsenicals and an antimonial. Drug sensitivity is represented as EC_50_ averages of 3–5 independent Alamar Blue experiments (means ± standard error of mean). The differences in the EC_50’_s of arsenic oxide, sodium arsenite and phenylarsine oxide (PAO) and potassium antimony tartrate (PAT) between *T. brucei*, *T. evansi* and *T. equiperdum* are only marginal. (**B**) Effects of 3.3 µM melarsomine (upper frame) or 0.33 µM PAO (lower frame) on the absorbance of trypanosomes over time. Measurements of absorbance were carried out, taking readings every 5 min with a PHERAstar microplate reader at 750 nm, and presented as averages of a single experiment performed in triplicates. Upper frame: time to 50% lysis was 49.5 ± 0.4 min for *T. b. brucei*, 53.7 ± 0.19 min for *T. equiperdum* and 52.7 ± 0.6 min for *T. evansi*. Lines were calculated by non-linear regression using an equation for a sigmoid curve with variable slope (Prism 9). Lower frame: 50% lysis was at 50.2 ± 0.5 min, 47.0 ± 0.7 min, 41.5 ± 0.3 min and 47.6 ± 0.5 min for *T. b. brucei*, *T. congolense*, *T. equiperdum* and *T. evansi*, respectively. (**C**) Effects of melarsomine (MelCy) and PAO on TcoIL3000 and the same cells expressing either TbAT1 or TbAQP2. Bars represent the averages and SEMs of three independent experiments. *** *p* < 0.001; NS, not significant. (**D**) Like frame B, using IL3000 and IL3000 expressing TbAT1. The drug was added at 15 min (arrowheads); readings were taken every 2 min. Symbols are averages of triplicate determinations. (**E**) Like frame D except with IL3000 expressing TbAQP2.

In *T. brucei*, the sensitivity of melaminophenyl arsenicals, such as melarsomine, depends on TbAT1 and TbAQP2, which transport them [19,21,24,49]. However, this does not apply to non-melaminophenyl arsenicals, such as phenylarsine oxide (PAO) [18]. The significant differences observed between the sensitivity of *T. congolense* and the brucei group to melarsomine prompted us to investigate sensitivity to non-melaminophenyl arsenicals in order to establish whether the difference is related to the melaminophenyl pharmacophore or to arsenic per se. *T. congolense* did not show any significant differences (one way Anova, Prism 9) in sensitivity to arsenicals other than those of the melaminophenyl class, including arsenic oxide, sodium arsenite and PAO, nor to the antimonial compound potassium antimony tartrate (PAT), screened using the Alamar Blue assay (Figure 6A). This clearly shows that the resistance to melarsomine is specific to melaminophenyl arsenicals and not to all arsenicals or heavy metals.

The rate of lysis of *Trypanosoma* species incubated with melarsomine and PAO was measured as the decrease in cell absorbance over time [18,50], based on the reduction of cell motility and increased cell lysis, leading to reduced light scatter and absorbance in the cuvette. While PAO generally killed the four *Trypanosoma* species at a similar rate, melarsomine showed only a minimal effect on *T. congolense* but rapidly lysed *T. brucei*, *T. evansi* and *T. equiperdum* (Figure 6B).

Upon expression of either TbAT1 or TbAQP2, *T. congolense* became significantly more sensitive to melarsomine (*p* < 0.001) in three different clones for the heterologous expressions, whereas the EC_50_ value for PAO remained unchanged (*p* > 0.05) (Figure 6C). Expression of TbAT1 had the most profound effect on the melarsomine sensitivity of *T. congolense*, with an average 26.3-fold lower EC_50_ in the +TbAT1 clones compared to a 4.8-fold difference for the +TbAQP2 clones. These results were further confirmed using lysis experiments: even at the very high concentration of 10 µM, *T. congolense* IL3000 was not sensitive to melarsomine whereas absorbance declined to 50% in, on average, 33 min and 30 min for +TbAT1 and +TbAQP2 cells, respectively (Figure 6D,E).

## 3. Materials and Methods

### 3.1. Parasites and Cultures

The bloodstream form (BSF) *T. b. brucei* s427 [49], *T. evansi* AntTat 3/3 [51] and *T. equiperdum* [38] were cultured in HMI-9 medium (Life Technologies, Paisley, United Kingdom) supplemented with 10% heat-inactivated foetal bovine serum (FBS (PAA Laboratories, Linz, Austria)), 14 µL/L β-mercaptoethanol (BDH, Dorset, UK) and 3.0 g/L NaHCO_3_ (Sigma-Aldrich, Gillingham, Dorset, UK) and adjusted to pH 7.4. These cell lines were maintained at 37 °C in a humidified, 5% CO_2_ environment.

The BSF *T. congolense* TcoIL3000 and derived cell lines were cultured in TCBSF3 medium without red blood cells. Dulbecco’s Minimum Essential Medium (MEM) was supplemented with 25 mM HEPES, 26 mM NaHCO_3_, 5.6 mM D-glucose, 1 mM sodium pyruvate, 40 μM adenosine, 100 μM hypoxanthine, 16.5 μM thymidine and 25 μM bathocuproinedisulfonic acid disodium salt. To this basal medium were added β-mercaptoethanol (0.0014% *v*/*v*), 1.6 mM glutamine, 100 units/mL penicillin/0.1 mg/mL streptomycin (Gibco), 20% goat serum (Gibco) and 5% Serum Plus (SAFC Biosciences) [52,53]. The BSF *T. congolense* IL3000 WT and its derived lines were cultured at 34 °C in a humidified, 5% CO_2_ environment. 

### 3.2. Resazurin-Based Drug Sensitivity Assay

The resazurin-based (Alamar Blue) drug sensitivity assay was used to assess and compare the activity of standard trypanocides in the different cell lines investigated [52,54]. Briefly, 23 doubling dilutions of the test drug starting at 100 μM plus drug free control were prepared in 100 μL medium in a 96-well white plate (Greiner Bio-one, Frickenhausen, Germany). The cells were adjusted to 2 × 10^4^ cells/mL for *T. b. brucei* and *T. equiperdum*; 4 × 10^4^ cells/mL for *T. evansi;* and 5 × 10^5^ cells/mL for *T. congolense* in the appropriate medium. Then, 100 μL of the adjusted cells was added to the wells containing drug dilutions and incubated for 48 h under respective culture conditions. Thereafter, 20 μL of 125 mg/mL resazurin sodium dye was added, and the plate was incubated for a further 24 h. The resorufin fluorescence in each well of the plate was determined and used to calculate the EC_50_ of the drug as described [42].

### 3.3. Growth Rate by Cell Count

A manual cell count of the cultures was carried out to determine the effect of expression of *T. brucei* transporters on the growth rate of *T. congolense*, as described [42]. Cell lines were counted and adjusted to the same density in 2 mL TCBSF3 medium and incubated in a 24-well plate. The density of each culture was determined by daily manual cell count using a Neubauer cell chamber and a phase-contrast light microscope (Zeiss). 

### 3.4. Cell Absorbance Assay

The rate of drug-induced lysis of trypanosomes was measured as a decrease in absorbance over time [18,50]. Cells were washed in assay buffer (AB; 33 mM HEPES, 98 mM NaCl, 4.6 mM KCl, 0.5 mM CaCl_2_, 0.07 mM MgSO_4_, 5.8 mM NaH_3_PO_4_, 0.03 mM MgCl_2_, 23 mM NaHCO_3_, 14 mM D-glucose, pH 7.3) and resuspended to 10^7^ cells/mL density in AB, after which 200 μL of the prepared cells was distributed into each well of a transparent-bottom black 96-well plate (Greiner Bio-one, Frickenhausen, Germany). Absorbance in each well was determined every 2 min using a microplate reader (PHERAstar or FLUORstar Optima (BMG Labtech, Durham, NC, USA)) set at 750 nm absorbance wavelength. At 15–20 min, 20 μL of either assay buffer or test drug at 10× the desired concentration in assay buffer was added to the respective wells and the measurements continued.

### 3.5. Immunofluorescence Microscopy

Immunofluorescence microscopy, carried out to visualise the localisation of the TbMFS transporter in *T. congolense*^TbMFT^, was performed as described previously [55]. MitoTracker Red^TM^ (Thermo Fisher Scientific, Hemel Hempstead, UK.) was added to the cell culture to a final concentration of 100 nM and incubated for 10 min. Culture containing approximately 2 × 10^6^ cells was collected in an Eppendorf tube, washed in 1× PBS and applied to a 12-well glass slide (Menzel-Gläser, VWR, Lutterworth, Leicestershire, UK) treated with Poly-L-Lysine (Sigma-Aldrich). After the cells were fixed with 4% paraformaldehyde and washed twice in 1× PBS, the slide was treated with 1% triton X-100 (Thermo Fisher Scientific) in PBS for 10 min and then with 100 mM glycine for 20 min, followed by a wash with PBS. The slide was blocked for 1 h with 1% BSA/0.2% Tween-20 (Sigma-Aldrich) and then treated with the primary antibody (rabbit anti-HA (Sigma-Aldrich)) at a dilution of 1:1000 in blocking solution and incubated in a wet chamber for 1 h. The sample was washed three times with PBS; 1:2000 secondary antibody (goat anti-rabbit IgG FITC conjugate (Sigma-Aldrich)) in blocking solution was added and incubated in the dark for 1 h. The slide was then washed three times with PBS, treated with DAPI and covered with a cover slip [55]. The prepared slide was viewed under an Olympus IX71 DeltaVision Core System (Applied Precision, GE Healthcare, Amersham, UK) using the SoftWoRx suite 2.0 software (Applied Precision, GE). All images acquired were processed using Fiji software [56].

### 3.6. Drug Uptake Assay

An uptake assay using radiolabelled trypanocides was carried out as described previously [57]. Radiolabelled ring-[^3^H]-DA was custom-made by PerkinElmer (CUST78468000MC; 60.7 Ci/mmol) and [^3^H]-pentamidine isethionate was custom-made by Amersham (TRQ40084; 3.26 TBq/mmol); [^3^H]-Suramin was supplied by American Radiolabeled Chemicals (ARC). Briefly, parasites were harvested washed twice by centrifugation in assay buffer before they were adjusted to a density of 1 × 10^8^ cells/mL in AB. Then, 100 µL of AB containing 1 × 10^7^ cells was mixed with the same volume of the radiolabelled drug in assay buffer, layered on top of an oil mix in microcentrifuge tubes and incubated for a pre-determined period. Uptake was stopped by the addition of high concentrations of ice-cold unlabelled substrate (stop solution) and immediate centrifugation of the cells through the oil layer. The microcentrifuge tubes were flash frozen in liquid nitrogen, and the bottom of each tube containing the cell pellet was cut off and transferred to a scintillation vial. Cell pellets were lysed with 2% SDS solution under slow agitation on a shaker for 30 min before the addition of 3 mL of scintillation fluid (Scintilogic U, Lablogic) into each vial and further incubation overnight in the dark. Radiation was measured in a 300SL (Hidex) scintillation counter (Hidex). Linear regression analysis using Prism (versions 8 and 9, GraphPad Software) allowed for the rate of uptake per unit time and other parameters, such as r^2^ and F-test, to be determined. For the determination of the *K_m_*, the radiolabelled permeant in the presence or absence of different concentrations of potential inhibitors reconstituted in assay buffer was placed on top of 250 μL oil mix in microcentrifuge tubes. 

### 3.7. Genetic Manipulation of T. congolense

Information for the genes investigated in this study, such as the nucleotide sequences, synteny and similarity to other genes, was obtained from the GeneDB (genedb.org) and TritrypDB (tritrypdb.org/tritrypdb) genome databases. The CLC Genomics Workbench 7 software (CLC Bio, Qiagen) was used for the design of primers, construction of plasmids and alignment of nucleotide sequences.

A modified pRM481 plasmid [58] named pMPB-DP-012, which targets the tubulin locus of *T. congolense* and transcribes blasticidin S deaminase (BSD) and offers a 6×HA tag at the C-terminal [59], was kindly supplied by Prof. Michael Barrett, University of Glasgow. The open reading frame (ORF) of *TbAQP2* was PCR-amplified from *T. brucei* genomic DNA and ligated into pMPB-DP-012 using *Sal*I and *Bam*H1, as described in [47,54] (primers detailed in Supplemental Table S1).

The *Sal*I site in the pMPB-DP-012 plasmid was replaced with a *Bgl*II site using the Q5 Site-Directed Mutagenesis Kit (NEB) as per the manufacturer’s protocol. Mutation in the generated plasmid was confirmed using PCR and Sanger sequencing (Source BioScience, Nottingham, UK). The correctly mutated plasmid was digested with *Bgl*II and *Bam*H1, followed by the insertion of amplified and purified *TbAT1* or lysosomal *TbMFST* using the NEB Builder HiFi DNA assembly cloning kit (New England Biolabs, Hitchin, Herts, UK.

Each plasmid was verified by Sanger sequencing for correct integration of the insert, linearised with *Asc*I (NEB, Hitchin, UK) and transfected into *T. congolense* IL3000. Transfection was carried out as described previously [60], after which 2–3 × 10^7^ cells of *T. congolense* IL3000 resuspended in transfection buffer were mixed with 10 µg of digested plasmid and electroporated using an Amaxa Nucleofector with the Z-001 programme. The transfected cells were transferred into 25 mL pre-warmed TC-BSF3 medium and then distributed into 24-well plates by limited dilution (1:5 and 1:10), followed by incubation for 18 h at 34 °C and 5% CO_2_ to allow for recovery. Cells were selected by the addition of 0.5 µg/mL blasticidin antibiotic to the growth medium, and the presence of the construct was confirmed by PCR amplification from the genomic DNA.

## 4. Conclusions

In this study, we have investigated the differences in sensitivity to standard trypanocides among several important species responsible for animal African trypanosomiasis worldwide. We found that the sensitivity of *T. b. brucei*, *T. evansi* and *T. equiperdum* (all *Trypanozoon* subgenus) to these drugs was generally very similar, with the exception of *T. b. brucei* and *T. evansi*, which are substantially less sensitive to ethidium bromide than *T. evansi*. However, *T. congolense* (subgenus *Nannomonas*) was clearly less sensitive to several important trypanocides, including suramin, melarsomine and the diamidines pentamidine and diminazene. The fact that *T. congolense* lacks orthologues for the known drug transporters for melaminophenyl arsenicals and diamidines (TbAT1 and TbAQP2), as well as the orthologue for the suspected lysosomal suramin transporter MFST, appears to give a rationale for the large differences in drug efficacy. TbAT1 is one of at least a dozen *T. brucei* purine transporters [61] and is not essential (although purine uptake is), with the gene easily deleted [25] and defective mutant alleles reported in field isolates [39,62]. In *T. congolense*, purine nucleosides are salvaged by the transporter known as TcoAT1, which is the orthologue of TbNT10, a broad selectivity purine nucleoside transporter [47] and, presumably, by a number of nucleobase transporters that are found in the genome [63] but not yet characterised. TbAQP2 is not essential either, in vitro or in vivo [21,39,64]. Indeed, TbAQP2 is a mutant copy of the conserved TbAQP3 [19,20], which has at least one orthologue in *T. congolense* (TcIL3000_10_12040). The function of TbMFST is not known, but its knockdown by RNAi suggests that it is not essential [28].

Expression of each of these transport proteins in *T. congolense* did sensitise this species to the various drugs except suramin. It also increased uptake of [^3^H]-pentamidine, [^3^H]-diminazene and the rate of cell lysis caused by melarsomine. We propose that the presence of absence of the TbAT1 and TbAQP2 transporters in various trypanosome species is a major determining factor of the extent to which a given species is sensitive to diamidines and melaminophenyl arsenicals, separating the *Trypanozoon* subgenus from subgenus *Nannomonas*. For suramin, the issue is more complicated, due to the higher complexity of its uptake mechanism, and was not resolved by the expression of *T. brucei* MFST. As such, the paradox of rapid suramin uptake and low suramin sensitivity in *T. congolense* will require further research. This research also reemphasises the need for animal trypanocides to be tested on multiple species, from different sub-genera.

## Figures and Tables

**Table 1:** Orthologues of *T. b. brucei* drug transporters, showing % identity by amino acid sequence.

Species	Gene ID	*T. brucei*		
		***AT1***		
			**Tb927.5.286b**	
*T. evansi*	AGT37292.1		99.35	
*T. equiperdum*	SCU70586.1		99.78	
*T. congolense*	-		<75	
		***AQP2***		
			**Tb927.10.14170**	
*T. evansi*	TevSTIB805.10.14910		100	
*T. equiperdum*	not annotated		100 ^1^	
*T. congolense*	-		<75	
		**MFST**		
		**Tb927.9.6360 ^2^**	**Tb927.9.6370**	**Tb927.9.6380**
*T. evansi*	TevSTIB805.9.4540	99.58	89.15	96.87
	TevSTIB805.9.4550	89.79	98.30	90.43
	TevSTIB805.9.4560	96.24	88.72	98.96
*T. equiperdum*	RHW70022.1	96.24	88.72	98.96
	RHW70658.1	88.30	97.23	90.21
	RHW70215.1	96.45	88.94	99.16
*T. congolense*	-	<75	<75	<75

^1^ P. Buscher and N. van Reet, personal communication; ^2^ Gene ID identified by Alsford et al. [28] as being associated with suramin resistance after RNAi knockdown.

## Data Availability

Not applicable.

## Supplementary Materials

The supporting information can be downloaded at: https://www.mdpi.com/article/10.3390/ijms23052844/s1.
