# Supplementary material for: Differences in Transporters Rather than Drug Targets Are the Principal Determinants of the Different Innate Sensitivities of Trypanosoma congolense and Trypanozoon Subgenus Trypanosomes to Diamidines and Melaminophenyl Arsenicals
Source: Int J Mol Sci. 2022 Mar 5;23(5):2844. doi: 10.3390/ijms23052844 (PMC8911344; doi:10.3390/ijms23052844)
Supplement: Supplementary file 1 [file ijms-23-02844-s001.zip › ijms-1621230-supplementary.pdf]

Table S1. List of primers

Primer	Primer sequence	Function
HDK1575	GTCGACATGCAGAGCCAACCAGAC	Forward primer for TbAQP2
HDK1576	GGATCCTTAGTGTGGAAGAAA	Reverse primer for TbAQP2
HDK1773	CAAAAGAAATACCACTTTCGCTACAAGATCTATGCTCGGGTTTGACTCA	Forward primer for TbAT1
HDK1774	CATAATCCGGTACATCATACGGATAGGATCCCTTGGGAAGCCCCTCATTG	Reverse primer for TbAT1
HDK1775	CAAAAGAAATACCACTTTCGCTACAAGATCTATGAGCAGCACAGACAATG	Forward primer for TbMFST
HDK1776	CATAATCCGGTACATCATACGGATAGGATCCAGTTCGTGATTTCGCTTTTGC	Reverse primer for TbMFST
HDK1573 [a]	TTTCGCTACAagatctATGAAAGTTATATCGTAC	Mutagenesis of pHDK273 plasmid
HDK1574	GTGGTATTTCTTTTGGTAAATCG	Mutagenesis of pHDK273 plasmid
MBP119	CATCCTCAAACTACTTACTCCG	Forward primer for pMB-DP-012 plasmid
MBP120	GCTTGTGCTGATACCTGGAG	Reverse primer for pMB-DP-012 plasmid

[a] lower case bases indicate the site of the intended mutation
